# Vascular Endothelial Growth Factors Enhance the Permeability of the Mouse Blood-brain Barrier

**DOI:** 10.1371/journal.pone.0086407

**Published:** 2014-02-14

**Authors:** Shize Jiang, Rui Xia, Yong Jiang, Lei Wang, Fabao Gao

**Affiliations:** 1 Molecular Imaging Laboratory, Department of Radiology, West China Hospital, Sichuan University, Chengdu, Sichuan, China, People’s Republic of; 2 Department of Pathology, West China Hospital, Sichuan University, Chengdu, Sichuan, China, People’s Republic of; Washington University, United States of America

## Abstract

The blood-brain barrier (BBB) impedes entry of many drugs into the brain, limiting clinical efficacy. A safe and efficient method for reversibly increasing BBB permeability would greatly facilitate central nervous system (CNS) drug delivery and expand the range of possible therapeutics to include water soluble compounds, proteins, nucleotides, and other large molecules. We examined the effect of vascular endothelial growth factor (VEGF) on BBB permeability in Kunming (KM) mice. Human VEGF165 was administered to treatment groups at two concentrations (1.6 or 3.0 µg/mouse), while controls received equal-volume saline. Changes in BBB permeability were measured by parenchymal accumulation of the contrast agent Gd-DTPA as assessed by 7 T magnetic resonance imaging (MRI). Mice were then injected with Evans blue, sacrificed 0.5 h later, and perfused transcardially. Brains were removed, fixed, and sectioned for histological study. Both VEGF groups exhibited a significantly greater signal intensity from the cerebral cortex and basal ganglia than controls (P<0.001). Evans blue fluorescence intensity was higher in the parenchyma and lower in the cerebrovasculature of VEGF-treated animals compared to controls. No significant brain edema was observed by diffusion weighted MRI (DWI) or histological staining. Exogenous application of VEGF can increase the permeability of the BBB without causing brain edema. Pretreatment with VEGF may be a feasible method to facilitate drug delivery into the CNS.

## Introduction

The blood-brain barrier (BBB) is a dynamically regulated physical barrier between the CNS and circulation consisting of endothelial cells (ECs) that line cerebral microvessels [1], [2]. While the BBB sustains the unique chemical microenvironment critical for neuronal activity in the CNS, it also restricts access to therapeutic drugs. These ECs are distinct from peripheral ECs in that they have a continuous basement membrane with no fenestrae and low pinocytotic activity [2]. Furthermore, complex tight junctions between adjacent ECs further restrict the diffusion of blood-borne substances. It has been estimated that almost all large-molecule drugs and more than 98% of small molecule drugs cannot cross the BBB. Rather, only small lipid-soluble molecules demonstrate significant BBB permeability [3]–[5].

Despite recent advances in CNS drug delivering methods [6]–[10], the safe and noninvasive delivery of many potential therapeutics remains a challenge [3]. Indeed, many potential therapies with demonstrated in vitro activity have proven ineffective in vivo due to lack of BBB permeability [11]. It is estimated that neurological disorders constitute at least 6.3% of the global burden and this burden is increasing [12]. However, most of the effective CNS drugs target only three types of disease: affective disorders, chronic pain, and epilepsy [5]. Many chemotherapeutic agents have shown favorable activity in *in vitro* models of neurological diseases, including Alzheimer’s disease, Parkinson’s disease, Huntington’s disease, and multiple sclerosis, but all have failed in clinical trials due to poor BBB permeability and failure to reach effective concentrations at appropriate CNS targets [13]. It has been estimated that only 12% of all drugs are active in the CNS, and only 1% are active against diseases other than affective disorders [14]. Furthermore, most traditional CNS drug delivery methods disrupt the BBB in both directions, resulting in side-effects and even permanent neural damage [14], [15]. For example, results of trans-cranial CNS drug delivery have been disappointing, and breaching the BBB has been associated with significant adverse events, including edema and leukocyte infiltration [14]. Although some noninvasive and specific approaches have been developed, it may take years before these techniques can be applied clinically in humans [16]–[18]. Thus, despite significant advances in our understanding of neurological disease pathogenesis, these disorders will remain a serious global health burden. So, it is of vital importance to find a safe and effective way to modulate the permeability of BBB to facilitate the entry of therapeutic drugs into the CNS for the treatment of neurological diseases.

The vascular endothelial growth factors (VEGFs A−D and multiple slice variants) induce vasculogenesis and angiogenesis during development, wound healing, and tumor growth [19]. Folkman *et al* [20] and Ide *et al* [21] found that tumors secrete molecules that promote angiogenesis with increased permeability. In 1989, VEGF was purified, cloned, and shown to be an effective endothelial cell mitogen [22], [23]. Exogenous application of VEGF reduced ischemic damage in an animal model of stroke [24]. However, the effects of VEGF may depend on when it is administered [25]. In addition to neuroprotection mediated by angiogenesis [21], [22], VEGF can also upregulate expression of the glucose transporter-1, thereby increase glucose transport across the BBB [26]–[28]. These studies suggest that VEGF may be used non-invasively to selectively modulate BBB permeability for drug delivery, but VEGF treatment protocols that optimize drug delivery while reducing the risk of hemorrhage must be developed. To this end, we measured BBB permeability following venous injection of different concentrations of VEGF in healthy mice and assessed ensuing edema.

## Materials and Methods

### Animal Procedures

Kunming (KM) mice (18−22 g) were purchased from the Chengdu Dossy Experimental Animals Co., Ltd (Chengdu, China) and treated according to the Guide for the Care and Use of Laboratory Animals issued by the US National Institute of Health (NIH Publication NO. 85–23). The experimental procedures were approved by the Care and Use of Experiment Animals Committee of Huaxi Medical Center, Sichuan University, Chengdu, China.

Animals were divided into two groups, one for determining the optimal time of VEGF administration and the second for determining the optimal VEGF injection concentration. The first group was pretreated with VEGF (8 µg/ml×200 µl, based on our preliminary study) by venous injection into the lateral vein and then examined by MRI at various times thereafter. The second group was divided into three subgroups and then treated with saline, low-dose VEGF (8 µg/ml×0. 2 mL = 1.6 µg), or high-dose VEGF (15 µg/ml VEGF×0. 2 mL = 3.0 µg), studied by MRI and then sacrificed for histological analysis.

### MRI Measurements

Magnetic resonance imaging was performed using a Bruker BioSpec 7 T/30 cm system (Bruker, Ettlingen, Germany). Images were recorded using a volume coil (outer diameter 44 mm and inner diameter 23 mm). Sequences including RARE-T2 [repetition time (TR) = 3000 ms; echo time (TE) = 45 ms, slice thickness 1 mm, Matrix 256×256, and a 18 mm field of view (FOV)], MSME-T1 (TR = 300 ms, TE = 11 ms, slice thickness 1 mm, matrix 256×256, FOV 18 mm), and DWI (TR = 3000 ms, TE = 30 ms, slice thickness 1 mm, matrix 128×128, FOV 25 mm). For DWI, the diffusion-weighting gradient was increased in a nonlinear manner from 0 to approximately 2.8 gauss/cm to obtain a series of six images with gradient b values of 100, 200, 400, 600, 800, and 1000 s/mm^2^. Number of diffusion direction 1. After a predetermined delay between infusion of VEGF165 or saline, a 200 µL bolus of gadolinium diethylenetriamine penta-acetic acid (Gd-DTPA, Omniscan Gadodiamide; Nycomed Inc., Princeton, New Jersey, USA; 0.1 mmol/kg) was injected intravenously. Contrast-enhanced MR images were T1-weighted with spin-echo sequences (TR = 500 ms, TE = 30 ms) and acquired in multislice mode (twelve slices, 1-mm slice thickness), 18 mm FOV, and a 256×256 image matrix. Apparent diffusion coefficient (ADC) maps were generated using Paravision 5.0 software. The signal intensity was normalized to the baseline acquired before Gd = DTPA injection to minimize possible variations resulting from different signal gain adjustments.

**Figure 1:**
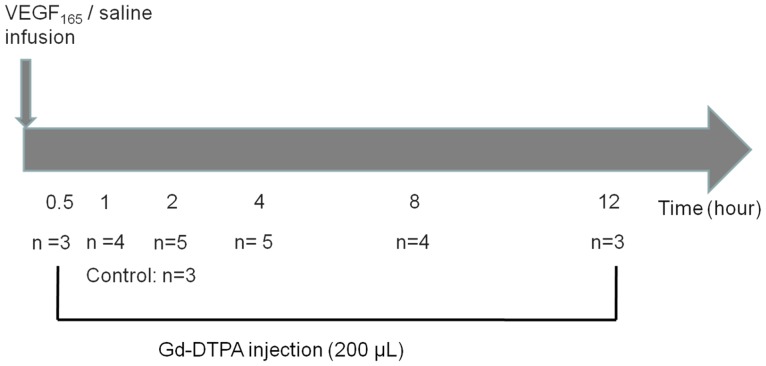
Schematic of the imaging protocol used to define the time window of the VEGF-induced increase in BBB permeability. The number of animals per subgroup is shown above.

Experiments determining the time window of changing BBB permeability were based on Marie *et al* [29] and were further refined in our experiment (Fig. 1). The contrast agent was administered 0.5, 1, 2, 4, 8, or 12 h after VEGF treatment. Based on these results, the delay between VEGF and Gd-DTPA injection in low- and high-dose groups was set at 8 h. Controls received an equal volume of saline. The animals were examined using MRI for at least 20 minutes after the injection of Gd-DTPA.

### Data Analysis

Regions of interest (ROIs) on T1-weighted images (precontrast and postcontrast) and DWI images included both cerebral hemispheres and basal ganglia. All ROIs were manually defined but were the same size and at approximately the same anatomical location from slice to slice. A water tube was place to the side of the animal and served as a reference.

### Statistical Analysis

One-way analysis of variance followed by a post-hoc t-test with Bonferroni correction was used for comparing MRI data among groups. A P-value less than 0.01 was considered statistically significant. Statistics tests were calculated using SPSS software (SPSS; SPSS, Chicago, Ill).

### Histology

After the MRI examination, 2% Evans blue in saline (3 ml/kg) was immediately injected into the tail vein. After 30 min, mice were deeply anesthetized with 10% chloral hydrate (3 ml/kg) and transcardially perfused with PBS until colorless fluid was observed coming from the right atrium. Thereafter, PBS was replaced by 4% buffered paraformaldehyde (PFA, pH 7.4). Animals from all groups were included in this analysis. Brains were quickly removed and placed in 4% PFA on ice. After 12 hours infusion in PFA, brains were washed five times in 0.01 M PBS (10 min/wash), coated with agarose (4%, Gene Company LTD, Chai Wan, Hong Kong), and sliced at 50 µm using a Leica VT 1000 S cryostat (Leica Microsystems, Wetzlar, Germany).

DAPI staining was performed as follows. The sections were soaked for 10 min in a solution of 0.1% DAPI (Beyotime Biotech, Jiangsu, China), drained, and washed three time in PBS (5 min/wash). Slides were air-dried, mounted with antifade medium (Beyotime Biotech, Jiangsu, China), and coverslipped before imaging under an Olympus BX51 fluorescence microscope (Olympus Corporation, Beijing, China) equipped with a QImaging® Retiga-2000 R digital camera (Microscope Services LTD, Oxford, UK). Images were analyzed with Image-Pro Plus 6.3 software (Media Cybernetics, Inc, Bethesda, Maryland). The differences in dye distribution between the brain parenchyma and blood vessels reflected the change in BBB permeability.

Paraffin sectioning and haematoxylin–eosin (HE) staining were performed as follows. At 24 hours after pretreatment with saline or VEGF165, animals were anesthetized and transcardially perfused as above. The brains were removed, fixed in PFA for 48 h at room temperature, blocked, dehydrated, cleared, infiltrated, embedded, sectioned at 5 µm, and stained. Two independent pathologists assessed slides for signs of brain edema.

## Results

The aim of this study was to enhance BBB permeability while minimizing damage by using the lowest doses of VEGF possible. In order to define the time window of enhanced BBB permeability after VEGF injections, we varied the delay between VEGF injection and administration of the contrast agent Gd-DTPA from 0.5 to 12 h (Figure 1). Signal intensity was elevated slightly when the delay was 2 h and increased to peak at 8 h before decreasing again. Thus, BBB permeability increases for at least 10 h after a 2 h delay.

To determine the concentration inducing the largest permeability increase without brain edema, mice were treated with saline (control Group A), 1.6 µg VEGF (low-dose Group B) or 3 µg VEGF (high-dose Group C) and BBB permeability and edema assessed 8−8.5 h later by MRI and histology. Figure 2.1 shows scans obtained before and 5 minutes after injection of Gd-DTPA for each group. Signal intensity enhancement caused by Gd-DTPA was apparent in brain while there was no difference in signal from muscle or skin. The signal intensity was significantly higher in both the low- and high-dose groups compared to the saline treatment group (Figure 2.1). The signal was more diffuse in the high-dose group, suggesting more extensive permeation of Gd-DTPA. Region of interest analysis indicated that signal was enhanced in both the cerebrum (P<0.001 for both dose groups compared to control; Figure 2.2) and basal ganglia (P<0.001; Figure 2.3) while there was no difference between VEGF-dose groups (cerebrum, P = 0.919; basal ganglia, P = 0.995).

**Figure 2:**
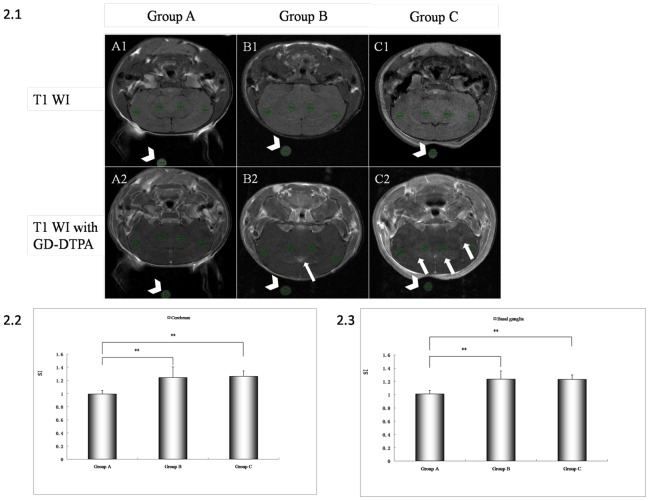
Changes in BBB permeability as revealed by MRI. Mice were pretreated with saline (Group A), 1.6 µg VEGF (Group B), or 3.0 µg VEGF (Group C) by venous injection 8 hours prior to administration of the contrast agent (Gd-DTPA). Mice from the three treatment groups were scanned before (Figure 2.1 A1, B1 and C1) and immediately after Gd-DTPA injection (Figure 2.1 A2, B2 and C2). Regions of interest (ROIs) was manually defined in both cerebral hemispheres and basal ganglia. ROI1 and ROI2 are located over regions of cerebral cortex and ROI3 and ROI4 over the basal ganglia. ROI5 is over the water tube. Compared to the saline-treated control group (Figure 2.1 A2), signal intensity enhancement was observed after treatment with 1.6 µg VEGF, particularly around cerebral ventricles (Figure 2.1 B2, arrow). Pretreatment with 3.0 µg VEGF also resulted in an obvious signal intensity enhancement in both cerebral cortex and basal ganglia (Figure 2.1 C2, arrows) comparable with saline treatment (Figure 2.1 A2). Arrow head indicates the water tube. (2.2, 2.3) Statistical analysis of signal intensity changes from the cerebrum and basal ganglia. Signal intensity values of each animal were calculated as follows: ROIa = (ROI1/ROI5+ROI2/ROI5)/2, ROIb =  (ROI3/ROI5+ROI4/ROI5)/2. ROIa and ROIb were used for the statistical analysis of the three groups. ROIa, signal intensity from the cerebral hemisphere. ROIb, signal intensity from the basal ganglia.

**Figure 3:**
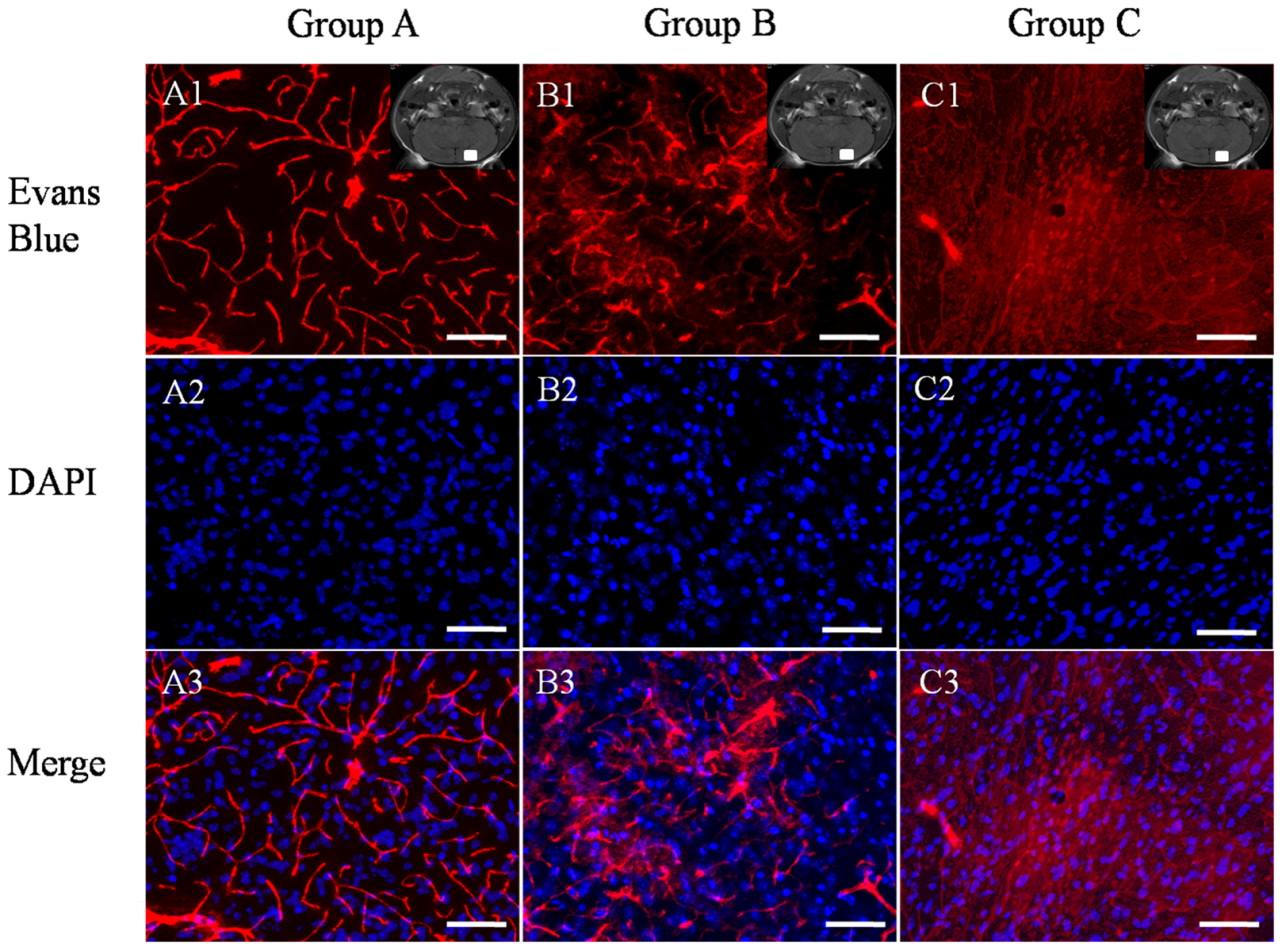
Permeability of blood vessels as revealed by Evans blue staining (2%, 3 ml/kg). Evans blue was injected 30 minutes before sacrifice. (A1) In the control group, red fluorescence was restricted to CNS blood vessels. (A2) By contrast, 8 hours after a single low dose of VEGF (1.6 µg), diffuse red fluorescence intensity was observed in the brain parenchyma as well as vessels. (A3) Pretreatment with 3.0 µg VEGF 8 hours prior to Evan blue injection resulted in more intense red fluorescence from the brain parenchyma, while the signal from blood vessels was reduced. Neural cells are DAPI-stained and images are merged. The white box in the top right corner of the MRI image indicates the location of the tissue used for histological study. Scale bar = 200 µm.

Mice were then injected with Evans blue and sacrificed for histological study (Figure 3). In the saline-treated control group A, red fluorescence was confined to blood vessels, indicating minimal breach of the BBB. In VEGF-treated mice, however, red dye was diffusely distributed in the neuronal parenchyma as well as in vessels, particularly in the high-dose group C.

It is known that increasing BBB can disrupt the neural microenvironment, with ensuing neuronal dysfunction. Indeed, disruption of the BBB is an early feature of lesion formation and leads to edema, entry of serum proteins, and inflammation. To assess edema, ADC maps were generated from DW-MR images while brain sections were examined by HE staining. There were no significant differences in cortical ADC values between control and either the lower concentration group (p = 0.284) or higher concentration group (p = 0.549) (Figure 4.2). Similarly, ADC values in the basal ganglia were not significantly different from controls in either the low concentration group (p = 0.583) or the high concentration group (p = 0.578) (Figure 4.3). Histological studies were conducted to confirm these DWI results. Again, there were no obvious visual signs of damaging parenchymal edema (Figure 4.4). However, small voids around small vessels were observed, suggesting mild edema, although these may be artifacts of the dehydration procedure for paraffin sectioning and HE staining.

**Figure 4:**
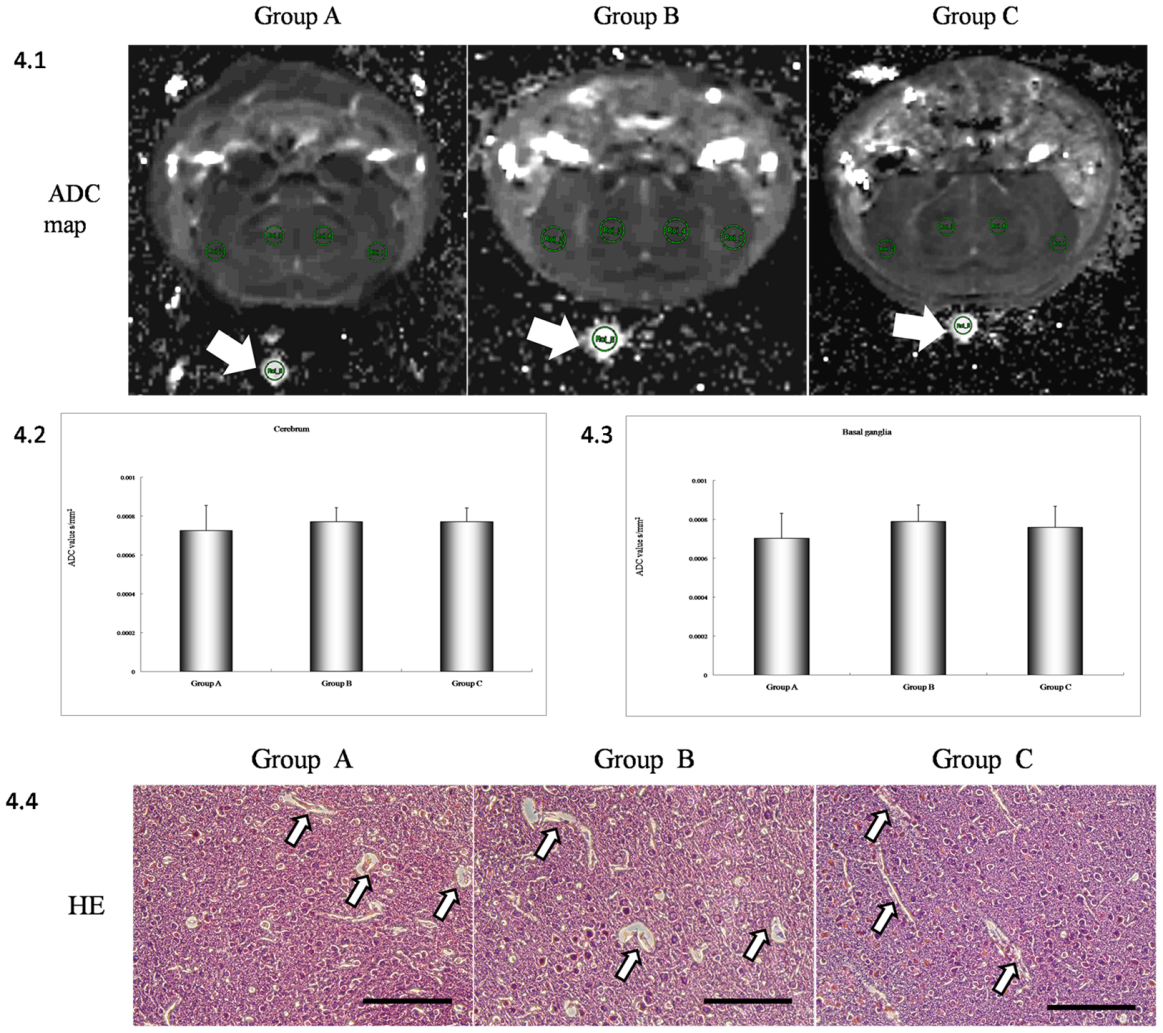
MRI and histological study of brain edema. (4.1) ADC maps for all three treatment groups. No obvious signs of edema were observed in cerebrum and basal ganglia. Arrow indicates water tube. (4.2, 4.3) Statistic analysis of ADC values from the cerebrum and basal ganglia. ADC values for each animal were calculated as follows: ROIc =  (ROI1+ROI2)/2, ROId =  (ROI3+ROI4)/2. ROIc and ROId were used for the statistic analysis. ROIc, ADC values of the cerebral hemisphere. ROId, ADC values of the basal ganglia. (4.4) Histological staining using HE. No obvious differences were detected among the three groups. Small voids were detected between the parenchyma and outer vessel wall (arrows). Scale barz = 100 µm.

## Discussion

Several methods have been developed to enhance CNS drug delivery, with varying degrees of success. In the current study, we reveal a potential non-invasive therapeutic approach for enhancement of BBB permeability to facilitate CNS drug delivery, systemic pre-injection of VEGF. This result is consistent with an in vitro study showing that VEGF dose-dependently and reversibly enhanced permeability through cultured endothelial cell layers [30]. In contrast, others have reported that systemic application of VEGF only increases the permeability of already compromised BBA. For example, VEGF increased the BBB permeability around the ischemic region of a mouse stroke model but not in the contralateral hemisphere [25]. However, our study demonstrates that VEGF can indeed enhance the permeability of the intact BBB in healthy mice *in vivo*. Moreover, this effect was observed at relatively low concentrations and was not associated with severe edema. These lower concentrations have also been shown to increase BBB permeability without induction of vascular proliferation [31], but continuous infusion led to retraction of astrocyte endfeet, monocyte infiltration, and neuroinflammation. We suggest that a single low dose of VEGF may transiently enhanced BBB permeability without disrupting neural homeostasis for safe CNS delivery of neurotherapeutics.

The absence of enhanced BBB permeability in some brain regions, which made MR images appear multifocal rather than homogeneous, may be due to the lag phase phenomenon, where local VEGF concentration fails to reach a threshold concentration due to its relatively brief plasma half-life. Previous studies also revealed that most VEGF receptors are located on the abluminal side of the BBB, which may limit access to systemic VEGF in some brain regions [32], [33].

While the identification of VEGF as an enhancer of BBB permeability represents the first step toward possible use for CNS drug delivery, much work remains to be done to elucidate the molecular mechanism. It was reported that VEGF can upregulate the mRNA expression of both VEGF and its receptors Flt-1 and Flk-1 in endothelial cells [34], possibly initiating a positive feedback cycle leading to enhanced BBB permeability. It is known that VEGF is a heparin-binding endothelial cell mitogen and angiogenic factor *in vivo* [35]. Since angiogenesis depends on the migration of endothelial and peripheral cells, sprouting of new vessels might be linked to the reconstruction of existing vessels. Tissue reorganization requires activation of matrix metalloproteinases (MMPs) and VEGF enhances MMP-9 activity of *in vivo* ischemia models and *in vitro* BBB models [36]. In addition, the endothelial nitric-oxide synthase (eNOS) pathway may also mediate transient opening of the BBB [37]. It was reported that systemic administration of the selective eNOS inhibitor cavtratin in mice abrogated VEGF-induced BBB disruption and protected against neurologic deficits in the MS model system. Previous studies also highlighted a role for the src-suppressed C-kinase substrate (SSeCKS) in the regulation of BBB permeability. SSeCKS decreased VEGF expression by downregulating AP-1 and stimulating expression of angiopoietin-1 [38]. The contribution of these signaling mechanisms obviously depends on their sensitivity to serum VEGF concentration and duration of receptor interaction. In this study, we used a single dose and VEGF has a relatively short half-life [39], so downstream mechanisms are restricted to those activated by relatively brief Flt-1/Flk-1 stimulation. Obviously, these are the pathways that should be targeted to induce a brief and reversible increase in BBB permeability for the safe delivery of CNS drugs.

It is known that exogenous application of VEGF can increase vascular permeability in peripheral organs, so there are potential risks for non-neurological adverse events, particularly in patients at high risk for hemorrhage. Neoplasia is also a major concern because of its association with neovasculation but this may be obviated by recently developed targeted delivery systems for VEGF [40], [41]. These systems may allow for delivery of high VEGF concentrations to specific regions, reducing the risk of CNS and peripheral hemorrhage. It may also be possible to specifically alter VEGF serum half-life by protein modification to further minimize side effects and maximize efficacy [41]. As for concerns about neoplastic diseases, there is no direct evidence that elevated VEGF concentration alone leads to neoplasms, although caution is still warranted. On the contrary, VEGF signaling can promote functional recovery from stroke in rats [42] but does not increase cancer risk in otherwise health mice [43].

We conclude that single, low-dose VEGF may facilitate CNS drug delivery through the BBB. In future studies, we will design a series of molecular probes of different sizes to monitor BBB permeability dynamically in vivo and determine the exact time window for drug delivery [44]–[46]. This study is a major step toward clinical translation of this VEGF protocol for improving the pharmacological treatment of brain diseases. There are, however, several limitations to this study. First, the experiments were conducted on healthy mice and certain diseases do significantly affect basal BBB permeability and the response to VEGF. Second, the precise molecular mechanisms for VEGF enhancement of BBB permeability were not determined. Further investigations are needed to more accurately define the relationship between VEGF dose and the change in BBB permeability.
